# Changes in Adult Lipid Profiles Following a 12-Week Physical Exercise Program at Wellness Centers, Primary Health Care Corporation, Qatar: A Retrospective Cohort Study With Pre-post Comparison

**DOI:** 10.7759/cureus.83580

**Published:** 2025-05-06

**Authors:** Anees A Alyafei, Aysha MA Hussein, Senda Amdouni, Sara Tariq Al Abdulla, Salam M Alkiswani, Hebah M Rbabah

**Affiliations:** 1 Wellness Programs, Preventive Health, Primary Health Care Corporation, Doha, QAT

**Keywords:** aerobic exercises, anthropometric measurements, lipid profile, physical exercise, primary health care, resistance exercise

## Abstract

Background

Dyslipidemia is a prevalent cardiovascular risk factor in Qatar, largely driven by sedentary lifestyles. Physical exercise (PE) is a cornerstone of non-pharmacological management; however, its integration into routine clinical practice remains limited. This study examined changes in lipid profiles among adults who participated in a structured 12-week PE program at wellness centers operated by the Primary Health Care Corporation (PHCC) in Qatar. Additionally, it explored correlations between lipid changes and sociodemographic and anthropometric parameters.

Methodology

This retrospective study included adults who completed a 12-week supervised PE program between January 2022 and December 2023 across seven PHCC wellness centers. Participants engaged in moderate-intensity aerobic and resistance exercises three times weekly. Only those who attended ≥85% of sessions and had complete pre- and post-intervention data were included. Data were extracted from electronic medical records and included lipid profiles (total cholesterol, triglycerides (TGs), low-density lipoprotein (LDL), high-density lipoprotein (HDL)) and anthropometric measures (weight, waist circumference (WC), body mass index (BMI), fat mass (FM)). Paired t-tests assessed pre-post differences, and Pearson’s correlation examined associations with age, gender, and anthropometrics.

Results

Among 739 participants (mean age = 48.75 ± 12.83 years; 74.56% female), significant reductions were observed in total cholesterol (from 4.99 ± 0.95 to 4.93 ± 0.77 mmol/L; p = 0.03) and TGs (from 1.26 ± 0.66 to 1.17 ± 0.48 mmol/L; p < 0.001). LDL and HDL changes were not statistically significant. Anthropometric parameters improved significantly, including reductions in weight (p < 0.001), BMI (p < 0.001), WC (p < 0.001), and FM (p < 0.001). Specifically, 31.96% of participants with borderline total cholesterol levels demonstrated a reversal to the normal range among the 97 cases assessed. Notably, 71.43% of the 42 patients reversed their borderline profiles to normal for TGs, LDL, and HDL. Correlation analysis revealed strong associations between lipid subtypes but weak or insignificant associations with age, gender, or anthropometric measures.

Conclusions

The 12-week structured PE program at PHCC wellness centers led to statistically and clinically meaningful improvements in lipid profiles and body composition. While lipid-lowering effects were modest for some parameters, the intervention effectively normalized borderline lipid levels in a significant proportion of participants. These findings support the integration of structured PE into routine primary care to manage dyslipidemia and reduce cardiovascular risk.

## Introduction

Dyslipidemia is a recognized major risk factor for cardiovascular disease globally and across the Middle East [1]. The worldwide prevalence of dyslipidemia in adults varies widely depending on definitions, population characteristics, and geographic region. According to a systematic review and global epidemiological analysis, the estimated prevalence of dyslipidemia among adults ranges from 20% to 80% globally, with significant variation across countries and regions [2].

In Qatar, an Arabic-speaking country in the Middle East, the rising prevalence is closely associated with shifts toward more sedentary lifestyles, rapid urbanization, and significant dietary changes in recent decades. Recent meta-analyses, published in 2025, estimate that approximately 26% of Qatari adults are affected by dyslipidemia, a figure that aligns with findings from other countries in the region and highlights a concerning upward trend in prevalence [3].

Physical exercise (PE) has been extensively studied for its ability to improve lipid profiles among adults. Still, it was not widely prescribed as an essential part of clinical practice, prevention, or management plans [4]. Although dyslipidemia is a major risk factor for cardiovascular diseases globally and in Qatar, it is primarily attributed to significant lifestyle changes over the last few decades. Data indicate that the progressive increase in the prevalence of dyslipidemia in Qatar underscores the urgent need for targeted public health strategies and interventions to address the growing burden of dyslipidemia and its related complications [5].

Muscella et al. identified significant health improvements resulting from regular PE, where aerobic exercises, such as running, and resistance exercises, such as push-ups, have been shown to positively impact lipid profiles [6]. Further confirmation by Wood et al. revealed that engaging in 12 weeks of aerobic exercise led to a 12% reduction in low-density lipoprotein (LDL) cholesterol, a 15% increase in high-density lipoprotein (HDL) cholesterol, and a 10% decrease in triglycerides (TGs) [7]. However, while aerobic exercise demonstrates improvements in HDL and TGs, its effect on total cholesterol is less substantial, as noted in some reviews, necessitating additional interventions for broader lipid management, as concluded by Smart et al. [8].

Although less impactful when used alone, resistance training showed marked improvements when combined with aerobic training, highlighting the complementary effects of multimodal exercise regimens. Combined programs yielded the most comprehensive lipid profile improvements, addressing total cholesterol, LDL cholesterol, TGs, and very-low-density lipoprotein cholesterol, as reported by Oh and colleagues [9].

Several studies have addressed the different predictors that influence the effect of PE on biochemical markers, including the lipid profile. The predictors may include sociodemographic factors, such as gender and age, as well as anthropometric measures, such as body mass index (BMI), waist circumference (WC), and fat mass (FM) [10,11].

Increasing age is usually associated with worsening lipid profiles, with increases in total cholesterol, LDL, and TG and decreases in HDL. However, regular physical activity, especially aerobic exercise, can mitigate these age-related changes by improving lipid profiles in older adults [12]. In contrast, Santos et al. reported that women often adhere less to PE programs than men, which resulted in less impact on the lipid profile [13].

The Primary Health Care Corporation (PHCC) is a leading governmental institute that provides primary care to the community in Qatar. A recently implemented wellness program initiative aims to promote behavioral modifications toward healthier lifestyles within the population through structured PE programs. In alignment with the management plan, seven wellness centers across Qatar have implemented PE training for adult patients who have been referred to participate in healthy lifestyle management. These centers conduct pre- and post-PE assessments, which include evaluations of each patient’s lipid profiles and anthropometric measurements. This process presents an opportunity to assess the impact of the post-PE evaluation.

The present study aimed to examine the changes of a structured 12-week PE program on adult lipid profiles, specifically total cholesterol, TGs, LDL, and HDL, measured in mmol/L, among participants at PHCC wellness centers in 2023. We hypothesized that engagement in this program would improve lipid parameters and body composition. Additionally, the study explored the correlation between lipid profile changes and participants’ sociodemographic characteristics and anthropometric measurements to identify potential predictors of response to PE.

## Materials and methods

Study design and setting

This retrospective cohort study with pre-post comparison utilized data acquired from the Electronic Medical Records System (EMRS) at the PHCC wellness centers from January 2022 through December 2023.

Study population

The participants in this study were adults who completed a 12-week PE training at the seven wellness centers in PHCC from January 2022 to December 2023.

Study intervention

The intervention consisted of a 12-week PE program implemented across seven wellness centers. This program included three sessions weekly, each lasting 60 minutes, which emphasized moderate-intensity exercise aimed at maintaining a heart rate above 70% of each participant’s maximum capability. The maximum heart capacity was determined using the maximum heart rate related to age (220 - age) [14]. Heart rate monitoring was conducted throughout the sessions, all of which were supervised by certified gym instructors to ensure consistency and standardization in delivery.

The PE program combined both aerobic exercises and resistance training with weights, structured to include phases for gradual warm-up, the main workout, and progressive cooldown. Adherence to the PE regimen was monitored through attendance records documented in the EMRS, with patients required to attend at least 85% of the scheduled sessions to be considered for the final analysis. Pre- and post-intervention evaluations were conducted for all patients, focusing on lipid profiles and anthropometric measurements.

Sampling strategy

A consecutive sampling methodology was utilized, incorporating all eligible patients who completed the program and had both pre- and post-intervention data accessible. This approach guaranteed that all qualifying patient data within the designated timeframe were included without the imposition of predetermined selection criteria.

Exclusion criteria

To maintain the integrity and validity of the results, we excluded patients who had incomplete data sets, erroneous data entries, or missing critical information pertinent to the study’s objectives. Furthermore, patients who had been prescribed weight management medications, including orlistat and tripeptide, or antihyperglycemic agents, such as metformin, insulin, or semaglutide, or those who had initiated or modified medical dietary regimens within three months before the intervention were also excluded from the analysis. This was done to minimize confounding factors and ensure that any observed changes in lipid profiles could be attributed primarily to the PE intervention.

Data collection

Data were meticulously gathered from the EMRS, focusing on adult patients, above 18 years of age, who successfully completed a rigorous 12-week PE program, along with their corresponding laboratory test results. The demographic information was documented, including age in completed years and gender (categorized as male or female).

The lipid profile parameters assessed included total cholesterol levels, TGs, LDL, and HDL, all expressed in mmol/L for each eligible patient. In addition to lipid measurements, comprehensive anthropometric data were extracted, including weight (kg), WC (cm), BMI (kg/m²), and FM (kg).

To ensure confidentiality and data integrity, the anonymized research data were securely stored within a password-protected Microsoft Excel (Microsoft Corp., Redmond, WA, USA) file, with access strictly limited to authorized members of the research team. This meticulous approach underscores our commitment to maintaining the highest standards of research ethics and data management.

Quality measures

The Institutional Review Board (IRB) at PHCC approved the study, ensuring compliance with corporate ethical guidelines. All data were de-identified to safeguard participant confidentiality. A data extraction protocol approved by the IRB was implemented to systematically identify and document data from electronic medical records (EMRs). The research team, inclusive of the principal investigator, underwent comprehensive training to ensure accuracy in the data collection process. Regular reliability checks were conducted by different team members to ensure uniformity in the evaluation of patient records, with any discrepancies resolved through consensus or further review by the principal investigator. Only complete and comparable datasets were analyzed, and routine audits in conjunction with validation procedures were employed to cross-reference the extracted data with EMR source documents, thereby ensuring accuracy and protocol compliance.

All anthropometric measurements, including body composition analysis utilizing calibrated bioelectrical impedance analysis machines and standardized stadiometers, were conducted in compliance with PHCC clinical practice guidelines, aligning with international standards. The calculations for BMI and WC thresholds adhered to World Health Organization guidelines [15]. Trained healthcare professionals at PHCC wellness centers executed anthropometric measurements using established protocols at baseline (pre-intervention) and at week 12 post-intervention.

Before referral to the wellness center, all patients were instructed to undergo standard blood tests, which included lipid profiles and other biochemical markers. All blood tests were conducted in laboratories affiliated with PHCC, utilizing validated biochemical assays.

The reference ranges for lipid profile parameters in mmol/L were defined as follows: total cholesterol: normal (<5.2), borderline (5.3-6.2), and high (>6.2); TGs: normal (<1.7), borderline (1.7-2.2), and high (>2.2); LDL: normal (<3.36), borderline (3.36-4.11), and high (>4.12); and HDL: normal (>1.0), with values below this threshold considered abnormal [16].

Data analysis

In this study, descriptive statistics were utilized to provide an overview of patients’ demographic data and their clinical characteristics before the intervention. Categorical variables, such as age groups and gender, were presented as frequencies and percentages, while continuous variables were calculated as means ± standard deviations. A table was generated to summarize the averages of the lipid profile parameters, including total cholesterol, TGs, LDL, and HDL, measured in mmol/L, along with body weight, WC, BMI, and FM.

The patients were further stratified based on their lipid profiles, distinguishing those with normal from those with borderline levels. To assess mean differences in lipid profiles, a paired t-test was performed to evaluate statistical significance. Additionally, box plots were created to illustrate the pre- and post-intervention distribution of lipid profiles. The percentage of patients whose lipid profiles normalized, moving from borderline to normal levels, was calculated as well.

Moreover, the relationship between demographic factors (age and gender) and anthropometric measurements, in addition to changes in lipid profile, was analyzed using Pearson’s correlation test. All statistical analyses were performed using SPSS Statistics for Windows, Version 29.0.2.0 (IBM Corp., Armonk, NY, USA), with the significance threshold set at a p-value of less than 0.05.

## Results

A total of 739 adults completed the 12-week PE program delivered across seven wellness centers out of a total of 841 participants’ records. The majority of participants were middle-aged females, with most individuals falling within the 31-65-year age group. Baseline assessments revealed the presence of a subset that exhibited borderline lipid profile levels, as defined by PHCC standards. Specifically, 97 (13.00%) participants had borderline total cholesterol levels. Borderline TGs were identified in 42 (5.68%) participants, 87 (11.77%) participants had borderline LDL levels, and only 38 (5.14%) participants demonstrated lower HDL levels, as shown in Table 1.

**Table 1:** Baseline characteristics of the patients before the 12-week physical exercise program (n = 739). SD: standard deviation; WC: waist circumference; BMI: body mass index; TGs: triglycerides; LDL: low-density lipoprotein; HDL: high-density lipoprotein

Category	n (%)	Mean ± SD
Age categories (years)		
18–30	73 (9.88%)	24.52 ± 3.45
31–65	609 (82.41%)	49.57 ± 8.86
˃65	57 (7.71%)	70.98 ± 4.95
Total	739 (100%)	48.75 ± 12.83
Gender		
Female	551 (74.56%)	-
Male	188 (25.44%)	-
Total	739 (100%)	-
Anthropometric measurements		
Weight (kg)	-	80.00 ± 14.71
WC (cm), males	-	103.98 ± 12.51
WC (cm), females	-	98.52 ± 13.04
BMI (kg/m²)	-	31.15 ± 5.39
Fat mass (kg)	-	33.91 ± 8.42
Lipid profile (mmol/L)		
Total cholesterol	642 (86.87%)	4.99 ± 0.95
TGs	697 (94.31%)	1.26 ± 0.66
LDL	652 (88.23%)	3.02 ± 0.80
HDL	701 (94.86%)	1.40 ± 0.31
Borderline		
Total cholesterol	97 (13.00%)	5.71 ± 0.27
TGs	42 (5.68%)	1.87 ± 0.13
LDL	87 (11.77%)	3.67 ± 0.22
HDL	38 (5.14%)	0.91 ± 0.08

**Figure 1:**
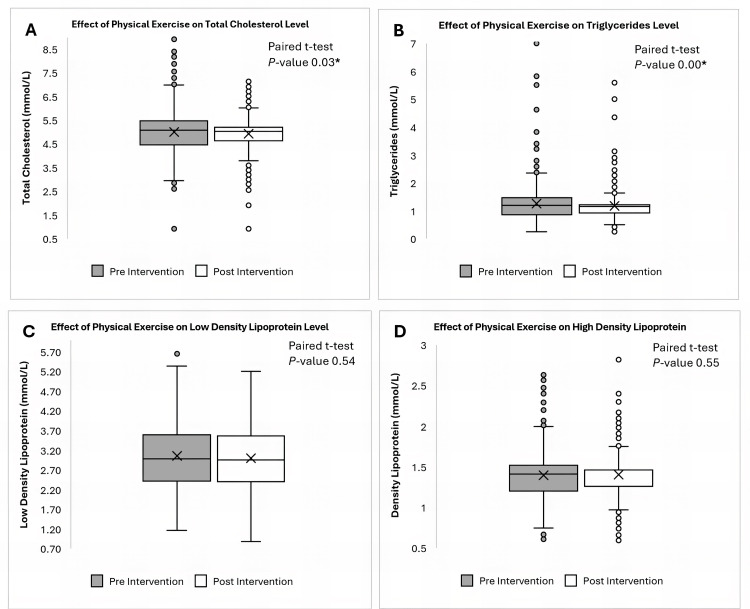
Box plot of changes in adult lipid profiles following a 12-week physical exercise program at wellness centers. (A) Box plot of the mean difference in total cholesterol (mmol/L). (B) Box plot of the mean difference in triglycerides (mmol/L). (C) Box plot of the mean difference in low-density lipoprotein (mmol/L). (D) Box plot of the mean difference in low-density lipoprotein (mmol/L).  Paired t-test. *: P-values <0.05 were considered statistically significant. *: A paired t-test revealed a statistically significant reduction in total cholesterol and triglyceride levels. At the same time, there was no statistically significant difference in either low-density lipoprotein or high-density lipoprotein levels following the physical exercise program (p < 0.05). Despite the presence of outliers, the observed change suggests an important effect of the intervention on the lipid profile.

Following the intervention, a favorable trend was observed across multiple lipid parameters. Figure 1 shows a box plot illustrating the change in the lipid profile. Total cholesterol levels decreased by a mean of 0.06 mmol/L, and TG levels showed a greater mean reduction of 0.09 mmol/L, with 31 out of 97 (31.96%) participants who had borderline total cholesterol and 30 out of 42 (71.43%) participants with borderline TGs shifting into the normal range. Notably, TGs exhibited the most substantial improvement among the lipid markers. Changes in LDL and HDL were more modest, with mean differences of 0.02 mmol/L and 0.01 mmol/L respectively; however, a subset of individuals still demonstrated meaningful improvements, as 30 out of 87 (34.48%) participants with borderline LDL and 14 out of 38 (36.84%) participants with low HDL levels achieved normalization post-intervention. These findings suggest a differential impact of the PE regimen, with more pronounced benefits observed among participants with borderline dyslipidemia at baseline (Table 2).

**Table 2:** Effect of the 12-week physical exercise program on lipid profile and anthropometric measurements. SD: standard deviation; TGs: triglycerides; LDL: low-density lipoprotein; HDL: high-density lipoprotein; WC: waist circumference; BMI: body mass index. Paired t-test. *: P-values <0.05.

Parameter	Mean ± SD, pre-intervention	Mean ± SD, post-intervention	Mean difference	Paired t-test (P-value)
Lipid profile				
Total cholesterol	4.99 ± 0.95	4.93 ± 0.77	0.06	2.15 (0.03)
TGs	1.26 ± 0.66	1.17 ± 0.48	0.09	4.34 (0.00)
LDL	3.02 ± 0.80	3.00 ± 0.65	0.02	0.61 (0.54)
HDL	1.40 ± 0.31	1.41 ± 0.27	0.01	-0.60 (0.55)
Anthropometric measurements				
Weight (kg)	80.00 ± 14.71	79.05 ± 13.89	0.95	0.000
WC (cm), males	103.98 ± 12.51	102.99 ± 12.22	0.99	0.010
WC (cm), females	98.52 ± 13.04	96.18 ± 13.12	2.34	0.000
BMI (kg/m^2^)	31.15 ± 5.39	30.88 ± 5.09	0.27	0.000
Fat mass (kg)	33.91 ± 8.42	32.49 ± 7.35	1.42	0.000

From an anthropometric perspective, the average body weight before the PE program was 80.00 ± 14.71 kg, while the average WC was 103.98 ± 12.51 cm for males and 98.52 ± 13.04 cm for females. The pre-intervention BMI was 31.15 ± 5.39 kg/m², and the average FM was 33.91 ± 8.42 kg. Body composition also improved in parallel, as evidenced by reductions in weight, BMI, and WC for females and males, and FM, with mean differences of 0.95 kg, 0.27 kg/m², 2.34 cm, 0.99 cm, and 1.42 kg, respectively. These anthropometric changes were more pronounced in females than in males, particularly in terms of WC reduction (Table 2).

The correlation matrix (Table 3), developed using Pearson’s correlation coefficient (r), revealed no significant associations between age or gender and changes in lipid profile parameters. This suggests that, within the studied population, variations in total cholesterol, TGs, HDL, and LDL levels following the intervention were not significantly influenced by either the participants’ age or gender.

**Table 3:** Correlation matrix between the changes in the lipid profile and age, gender, and anthropometric measurements. TGs: triglycerides; LDL: low-density lipoprotein; HDL: high-density lipoprotein; WC: waist circumference; BMI: body mass index. Pearson’s correlation coefficient (r).

	Age (years)	Gender (male/female)	Cholesterol (mmol/L)	TG (mmol/L)	HDL (mmol/L)	LDL (mmol/L)	Weight (kg)	WC (cm)	BMI (kg/m^2^)	Fat mass (kg)	Fat (%)
Age (years)	1										
Gender (male/female)	0.244	1.000									
Cholesterol (mmol/L)	-0.039	-0.061	1.000								
TG (mmol/L)	-0.038	-0.138	0.270	1.000							
HDL (mmol/L)	0.058	0.128	0.306	-0.199	1.000						
LDL (mmol/L)	-0.004	-0.070	0.862	0.100	0.125	1.000					
Weight (kg)	0.110	0.011	0.041	0.059	-0.018	0.012	1.000				
WC (cm)	0.055	0.045	-0.019	0.032	-0.032	-0.064	0.352	1.000			
BMI (kg/m^2^)	0.096	-0.008	-0.007	0.019	-0.019	-0.037	0.681	0.486	1.000		
Fat mass (kg)	0.015	0.019	-0.053	0.012	0.005	-0.084	0.349	0.253	0.368	1.000	
Fat (%)	-0.117	-0.271	0.015	0.060	-0.004	-0.008	0.123	0.134	0.184	0.384	1.000

In contrast, notable correlations were observed between lipid profile parameters and anthropometric measurements. A strong positive correlation was found between changes in total cholesterol and LDL (r = 0.86), indicating a parallel trend in their variation. Moderate correlations were also observed between total cholesterol and HDL (r = 0.31), and between cholesterol and TGs (r = 0.27), while HDL showed a weak negative correlation with TGs (r = -0.20).

However, the correlations between lipid parameters and anthropometric measures, including weight, WC, BMI, and FM, were generally weak and lacked clinical significance. These findings suggest that, although components of the lipid profile are interrelated, their changes after intervention were largely independent of changes in body composition.

## Discussion

This study aimed to evaluate changes in adult lipid profiles, specifically total cholesterol, TGs, LDL, and HDL, following the completion of a 12-week PE program at wellness centers operated by the PHCC in 2023. It also investigated the correlation between sociodemographic factors, anthropometric measurements, and changes in lipid profile.

The results support the effectiveness of the PE program in improving participants’ lipid profiles. These findings are consistent with those of Wood et al., who demonstrated that a 12-week aerobic intervention resulted in a 12% decrease in LDL, a 15% increase in HDL, and a 10% reduction in TGs [7]. Similarly, combined aerobic and resistance training programs have been shown to lead to significant improvements in lipid profiles, as noted by Jayedi et al. [17]. While aerobic exercise has a positive effect on TGs and HDL, its impact on total cholesterol is generally modest, underscoring the need for a combined approach to achieve optimal results [18]. While the absolute mean reduction was modest in total cholesterol, normalization occurred in 32% of borderline cases, indicating clinical relevance for at-risk individuals.

Age and gender were not potential contributing factors to the lipid profile changes observed in this study. With a mean age of 48.75 ± 12.83 years, the majority of participants were within the 31-65-year age group. ​Previous research has shown that younger individuals often exhibit greater physiological adaptability, leading to more favorable lipid profile responses following exercise interventions. For example, a study found that men with higher cardiorespiratory fitness levels retained more favorable lipid profiles from their early 20s to mid-70s, indicating that age and fitness levels significantly affect lipid metabolism [19].​ Although females made up 74.56% (551) of our study sample, existing literature presents mixed findings regarding gender differences in lipid metabolism in response to exercise. Some studies have observed that men might experience more pronounced improvements in certain lipid parameters, possibly due to differences in hormonal responses and exercise intensity. Conversely, other research has indicated that women tend to have higher HDL cholesterol levels and lower TG levels than men, suggesting more favorable baseline lipid profiles [20]. These discrepancies underscore the need for additional research to comprehensively understand the influence of gender on lipid profile responses to exercise.

Anthropometric improvements likely contributed to improvements in the lipid profile. Our study reported a pre-intervention mean BMI of 31.15 ± 5.39 kg/m² and FM of 33.91 ± 8.42 kg. Several studies, including those by Waked et al. and Zhou et al., emphasized that anthropometric changes following exercise correlate with favorable lipid metabolism, characterized by decreased TGs and elevated HDL [20,21].

Although BMI is widely used in clinical practice, emerging evidence suggests that other anthropometric indices, such as WC and lipid accumulation product, may offer greater predictive accuracy for metabolic disorders, including dyslipidemia [22]. However, not all studies support strong correlations between anthropometric measures and lipid profiles, indicating that individual variability and additional metabolic factors may influence these associations [23]. This underscores the complex interplay between obesity and lipid metabolism and highlights the need for further research to clarify these relationships.

However, this study is subject to several limitations. The absence of a control group prevents the definitive attribution of lipid profile changes solely to the PE program. Other unmeasured factors, such as dietary habits, medication adherence, or exercise outside the wellness centers, may have influenced the results. As this was a retrospective analysis relying on EMRS data, inclusion criteria depended on data completeness rather than randomization, which introduced potential selection bias. The absence of a control group in the retrospective design significantly undermines our ability to draw meaningful causal inferences. Moreover, the overrepresentation of women may impact the generalizability of the findings, especially given the recognized physiological differences in lipid response to PE between genders.

Despite standardized measurement protocols, variations in anthropometric assessments could introduce measurement bias. Future research should consider randomized controlled designs, longer follow-up periods to assess sustainability, and the inclusion of objective tools, such as wearable devices, to monitor PE adherence and intensity. Gender-balanced recruitment strategies would also strengthen subgroup analysis.

This study highlights the important role of structured PE in managing dyslipidemia and reducing cardiovascular risk. Utilizing real-world data from PHCC wellness centers enhances the findings’ relevance. While statistical significance was achieved, the clinical importance of changes in lipid parameters, especially for those at borderline or high risk, requires further exploration.

The improvements in lipid profiles following a 12-week PE program demonstrate the benefits of integrating exercise interventions into primary care. Given the high prevalence of non-communicable diseases in Qatar, expanding wellness services within the PHCC is essential for cardiovascular risk reduction. Public health policies should promote the inclusion of PE trainers in care teams and create referral pathways to PE programs. Additionally, implementing clinical decision-support systems and training healthcare providers in exercise prescription can make lifestyle interventions more effective. Culturally tailored educational campaigns are also vital for increasing patient engagement and awareness.

## Conclusions

This study highlights the positive impact of a structured PE program delivered in primary care wellness centers. The intervention was associated with favorable trends in lipid profiles and body composition, supporting its role as a practical, non-pharmacological approach for reducing the risk of chronic diseases. These findings underscore the importance of incorporating supervised exercise into routine clinical care to foster healthier lifestyles and enhance metabolic health outcomes. Moreover, the study suggests that individuals with borderline lipid levels may particularly benefit from such programs, with a notable proportion achieving normalization following the intervention. The improvements in anthropometric parameters further reinforce the broader metabolic benefits of regular PE. By leveraging the accessibility of wellness centers within the primary healthcare system, structured physical activity programs can serve as an effective tool in the prevention and early management of dyslipidemia and other non-communicable diseases. Future efforts should focus on scaling up such initiatives, integrating multidisciplinary teams, and ensuring sustained engagement to maximize long-term health outcomes. To further validate these findings, future studies employing randomized controlled trial designs are recommended to establish causality and assess long-term efficacy. A 12-week structured program of combined aerobic and resistance exercises improved lipid profiles, particularly TG levels. Integrating such programs into primary care with structured referrals and adherence support is recommended to enhance cardiovascular risk reduction in Qatar.
